# High-altitude populations need special considerations for COVID-19

**DOI:** 10.1038/s41467-020-17131-6

**Published:** 2020-07-01

**Authors:** Arnar Breevoort, Giovanni A. Carosso, Mohammed A. Mostajo-Radji

**Affiliations:** 1 The Eli and Edythe Broad Center of Regeneration Medicine and Stem Cell Research, University of California San Francisco, San Francisco, CA 94143 USA; 2 University of Amsterdam, Swammerdam Institute for Life Sciences, Science Park 904, Amsterdam, 1098 XH The Netherlands; 3 Clubes de Ciencia Bolivia Foundation, Santa Cruz de la Sierra, Bolivia; 4 Embassy of Science, Technology and Innovation, Ministry of Foreign Affairs of Bolivia, La Paz, Bolivia; 5 Permanent Mission of Bolivia to the United Nations, New York, NY 10017 USA

**Keywords:** Developing world, Policy

## Abstract

The atmospheric pressure that decreases with altitude affects lung physiology. However, these changes in physiology are not usually considered in ventilator design and testing. We argue that high altitude human populations require special attention to access the international supply of ventilators.

Humans are naturally adapted to live at low altitude. Yet, ~2% of the world’s population permanently live at altitudes above 2500 meters^1^. The majority of these populations live in areas that are either poor, such as Ethiopia, Ecuador and Bolivia, or highly controlled, such as Tibet^1^. The small market size and low economic power of these regions have left them aside in the design of biomedical equipment, which usually does not consider their special environmental conditions and physiological adaptations.

Worldwide, there is a shortage of many goods including intensive care unit (ICU) beds, facemasks, and ventilators due to the COVID-19 pandemic^2^. This shortage has led to an international “stock market”-like process of bidding for these goods, in which many countries are left at an extreme disadvantage^3^. Several international organizations, including the International Monetary Fund, the World Bank, and others, have been encouraged to provide loans to developing countries, but access to these goods is an issue that goes beyond money. Many countries have enacted export restrictions, and small orders are usually rejected to give preference to large purchases from powerful nations^3^. This reduces the capability of less-affluent nations in both treating COVID-19 patients and preventing the further spread of the disease.

Diverse pathological mechanisms in COVID-19 are under investigation, and respiratory symptoms predominate in the clinic. Physiologically, SARS-COV-2 cell entry is dependent on the cellular expression of ACE2 and TMPRSS2, and SARS-COV-2 likely binds to, and replicates in epithelial cells after entering the nasal cavity^4^. SARS-CoV-2 then spreads from the nasal cavity into the lungs where it primarily infects ciliated epithelial cells that line the conducting airways. As ACE2 expression and receptors are also found in cells outside of the lungs^5^ and COVID-19 patients have been reported to suffer from non-lung-related illness^6^, it is likely that SARS-CoV-2 infection is not limited to the nasal cavity and lungs. However, the majority of COVID-19 related deaths are caused by pulmonary illness. Therefore, the lungs are the primary focus of COVID-19 treatment efforts.

Ventilators are essential to treat COVID-19, as they are the primary equipment needed to assist the breathing and gas exchange of critically ill patients. To this end, ICU ventilators have become an expensive commodity that most countries have fought to access. These fights have left aside traditional international diplomatic conventions. Thus far, 54 countries have placed restrictions on the export of ICU ventilators^7^. Moreover, on several occasions countries have confiscated other countries’ ventilators during transit through their borders, which the press has referred to as “modern-day piracy”^8^.

Commercial ICU ventilators are largely designed and tested near sea level. Yet, with altitude there is a progressive reduction in barometric pressure and subsequently in oxygen pressure leading to major physiological adaptations in the lungs^9^, which are not usually considered in ventilator quality tests. Limited studies have compared the accuracy in tidal volume delivery of commercial ventilators at varying altitude and were all in the context of aeromedical evacuation^10,11^. In these tests, the majority of commercial ventilators failed at high altitude, delivering tidal volumes with up to 40% error from the set volume^10^. Importantly, technical errors and canceled ventilation were frequently reported^10^. Long-term care of critically ill patients, including COVID-19-positive patients, at high altitude is therefore unfeasible with the majority of these ventilators and there is a general lack of ventilators specialized for the 140 million people living at higher elevations.

History shows that a failure to contain a pandemic locally can lead to increased viral spread, and an increase in the global overall morbidity and mortality of a virus. The 1918 H1N1 pandemic and the 2019 SARS-COV2 pandemic are similar in which both the H1N1 and SARS-CoV-2 virus are antigenically novel, transferred zoonotically to humans, have adapted to the human body and are a cause of severe respiratory illness^12^. After the initial failure to contain the spread of H1N1 in 1918, a second more pathogenic wave swept across the world, accounting for the majority of casualties related to H1N1^12^. This second wave particularly affected ill-prepared populations which then became new sources of viral spread.

Adequate containment and treatment of a virus is paramount to prevent its spread and hamper potential increase in pathogenicity. It is therefore in the interest of the international community as a whole that each nation is able to strongly respond to COVID-19. Geographically, several high-altitude countries are located and well connected to other nations with high population density. Bolivia, for example, being at the center of South America, neighbors five countries, including Brazil, the most populated country in the region. Having one of the poorest healthcare systems in the Americas, it is common for Bolivians to search for medical treatment in neighboring countries^13^. Consequently, failure to contain and treat the virus in Bolivia would hamper the efforts of other South American countries. Similarly, failure to contain the virus in Ethiopia, the second most populated country in Africa, would negatively affect the treatment efforts of the region.

When international collaboration ensures that less-affluent nations are able to respond strongly locally, COVID-19 can be contained internationally. The West-African Ebola outbreak in 2014 met a strong international response spearheaded by the United States and the World Health Organization (WHO) resulting in the end of the internationally concerning public health emergency regarding Ebola in early 2016^14^. The further spread of COVID-19 can be halted under international collaboration and the understanding that success is dependent on the containment of COVID-19 in all countries, including economically challenged countries^15^. However, this success relies on two factors. Firstly, equipment to adequately treat and test for COVID-19 should be developed and made available that matches the geographical, economic, and educational challenges that are relevant to these countries. This is not limited to the development of ICU ventilators that are functional at higher elevation but can also be aimed at improving testing methods for COVID-19 at lower cost and easy scalability to a relatively small population of trained laboratorians. Secondly, although it is inherent that each country aims to acquire the necessary amount of equipment required for the treatment and prevention of COVID-19, less-affluent countries should receive international support in their efforts to obtain the equipment and tests that are necessary and relevant to their response to COVID-19. Implementation of these two factors will both improve the epidemiological response of economically and geographically challenged countries to COVID-19 in a humanitarian way and reduce the chance that these countries might become centers of new SARS-COV-2 outbreaks to neighboring countries.
